# Three-dimensional dynamic morphology of the mitral valve in different forms of mitral valve prolapse – potential implications for annuloplasty ring selection

**DOI:** 10.1186/s12947-016-0073-4

**Published:** 2016-08-15

**Authors:** Astrid Apor, Anikó Ilona Nagy, Attila Kovács, Aristomenis Manouras, Péter Andrássy, Béla Merkely

**Affiliations:** 1 Heart and Vascular Center, Semmelweis University, Gaál J.u.9, Budapest, H-1122 Hungary; 2 Department of Cardiology, Karolinska University Hospital, Stockholm, Sweden; 3 Bajcsy-Zsilinszky Hospital and Clinic, Budapest, Hungary

**Keywords:** Real-time three dimensional transesophageal echocardiography, Mitral valve dynamics, Myxomatous, Annuloplasty ring

## Abstract

**Background:**

Real-time three-dimensional transesophageal echocardiography has increased our understanding of the distinct pathomechanisms underlying functional, ischaemic or degenerative mitral regurgitation. However, potential differences in dynamic morphology between the subtypes of degenerative mitral prolapse have scarcely been investigated.

**Methods:**

In order to compare the dynamic behavior of the different phenotypes of degenerative mitral valve prolapse, real-time three-dimensional transesophageal echocardiography recordings of 77 subjects, 27 with Barlow disease (BD), 32 with Fibroelastic deficiency (FED) and 18 normal controls (NC) were analysed.

**Results:**

Geometric annular and valvular parameters of the myxomatous patients were significantly larger compared to controls (BD vs. FED vs. NC 3D annular area: 15 ± 2.8 vs. 13.3 ± 2.4 vs. 10.6 ± 2.3cm^2^, all *p* < 0.01). Beside similar ellipticity, BD annuli were significantly flatter compared to FED. Myxomatous annuli appeared less dynamic than normals, with decreased overall 3D area change, however only the BD group differed from NC significantly (BD vs. FED vs. NC normalized 3D area change 4.40 vs. 6.81 vs. 9.69 %; BD vs. NC *p* = 0.000; FED vs. NC *p* = not significant, BD vs. FED *p* = 0.025).

**Conclusion:**

BD and FED differ not only in terms of valve morphology, but also annular dynamics. Both pathologies are characterized by annular dilatation. However, in BD the annulus is remarkably flattened and hypodynamic, whereas in FED its saddle-shape and contractile function is relatively preserved. These features might influence the choice of repair technique and the selection of annuloplasty ring.

## Background

The development of 3D TEE technology revolutionized our understanding of the complex structure and function of the mitral apparatus. Appreciation of the importance of the dynamic 3D geometry of the mitral annulus (MA), responsible for minimizing leaflet stress and optimizing coaptation, reshaped our approach to mitral valve repair. A wide range of annuloplasty rings, based on individual concepts have been developed, including rigid, flexible, saddle shaped, and flat rings, with the intention to achieve more physiologic repair results from viewpoint of natural annular geometry and dynamics. Consensus regarding superiority of either of these products is still lacking. Data from studies investigating to which extent the hypothethized advantages of various rings manifest in reality remain contentious. Controversies in results might, at least in part, be attributable to differential alterations of the mitral apparatus in various pathologies.

Morphology and dynamics of the normal MA [1–4], as well as distinctive structural and functional alterations in various etiologies of mitral regurgitation (MR) including functional (FMR) and MR due to myxomatous degeneration have been described [5, 6], however, the limited data available on the dynamic behaviour of myxomatous annuli are contradictory. We hypothesized that BD and FED patients, who display remarkable phenotypic differences in valvular morphology, might also represent discriminative cohorts with respect to the geometric and functional characteristics of the mitral annulus. The aim of the present study was to investigate the geometric properties and the dynamicity of the MA out of special consideration for the different subtypes of myxomatous mitral valve disease, i.e. BD and FED, compared to healthy individuals.

## Methods

### Patient population

Between January 2010 and August 2012, 66 consecutive patients with moderate to severe degenerative MR were enrolled retrospectively. 20 control subjects (NC) referred for TEE (e.g. because of fever of unknown origin to rule out infective endocarditis or following a peripheral embolic event in order to rule a cardiogenic source of embolism) and found to have no relevant underlying structural heart disease were enrolled. Patients with permanent atrial fibrillation, concomitant other relevant valvular lesion, prosthetic valve, known ischaemic or rheumatic heart disease, history of infective endocarditis, or with cardiomyopathy of any etiology, were excluded. All subjects had preserved ejection fraction without regional wall motion abnormality. Standard 2D transthoracic (2DTTE) and RT3DTEE examinations were performed on the same day. Every patient signed an informed consent to participate in the study. TEE was performed during conscious awake sedation. Subjects with poor image quality that we considered would not have allowed reliable and reproducible measurements were excluded, resulting in 59 patients and 18 control subjects included in the final analysis. Patients were classified into BD or FED based on clinical presentation and the characteristic 2D and 3D echocardiographic findings described previously [7] resulting in 27 patients in BD and 32 patients in FED category.

### Echocardiography

An iE33 system (Philips Medical System, Andover, MA), equipped with S5-1 phased array and X7-2t matrix TEE transducers was used. Chamber dimensions and ejection fraction were calculated using the modified Simpson’s method [8]. The degree of MR was assessed by integrating indices of severity in accordance with the current guideline [9]. During RT3DTEE first 3D zoom, then ECG-gated full-volume data sets were collected over 4 cardiac cycles. The region of interest was set to the smallest possible volume to encompass the entire mitral apparatus resulting in frame rates of 17 to 35 frames/s in full volume acquisition. Data sets with stitching artifacts were excluded and loops of the best image quality were selected for analysis.

### Image analysis

**Fig. 1:**
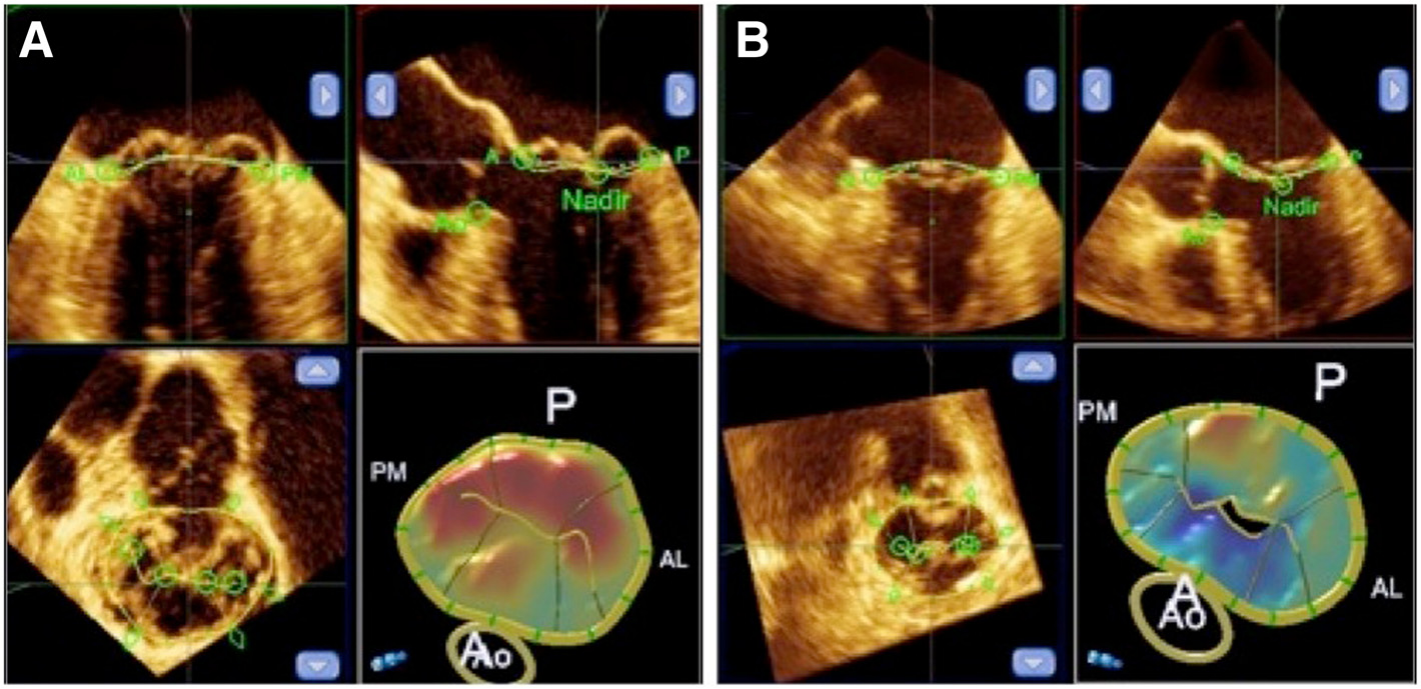
Static 3D model of the mitral valve in Barlow disease (**a**) and FED (**b**) produced by the Qlab software. Multiplanar reconstruction of the 3D data set, in the right lower corner color-coded, surface rendered model of the valve. Prolapsing scallops are color coded in red

Data sets were analyzed off-line by a single experienced cardiologist blinded to patient data. Static geometric parameters were measured at end-systole (defined as the last frame before MV opening) using QLAB Mitral Valve Quantification software (version 9.0, Philips Medical System). In a series of 2D projection planes generated from the 3D data set the annulus and leaflets were traced manually. After the delineation of the annulus and leaflets the software generated a 3D model of the valve and automatically measured the various parameters of annular and leaflet geometry. Inter alia anteroposterior (AP) and transverse annular diameters, ellipticity index (transverse to AP diameter ratio × 100), 3D minimal surface area, height and height to commissural width ratio (AHCWR) of the annulus, as well as the anterior to posterior leaflet non-planar angle, 3D surface area of the leaflets, and prolapse height and volume were calculated (Fig. 1a, b). Dynamic behavior of the MA was analysed using 4D-MV Assessment software 2.1 (Tomtec Imaging Systems, Munich, Germany), which creates a dynamic model of the valve throughout the systolic phase of the cardiac cycle, based on a speckle tracking algorithm. Several parameters, including 3D annular area, circumference, AP and transverse diameters of the annulus were measured automatically. 3D area and 2D (3D area in projection plane) area change fraction of the annulus served as a base for the estimation of annular contraction. For illustration and statistical analysis the dynamic data sets were time shifted, so that the beginning of systole became the first time point and normalized in time to provide uniform systolic periods for each patient. Continuous parameters were normalized for their end-systolic value. Spline interpolation was used to get uniform data before mean values and standard deviations were calculated. Relative changes of the measured parameters were calculated as the ratio of the minimal and maximal normalized value of the actual continuous parameter, and used for statistical analysis of the dynamic data sets. Intra-observer variability was assessed in 10 randomly selected patients; repeated measurements were performed at least one week after the initial measurement and the investigator was blinded to previous results.

### Statistics

SPSS version 16.0 (SPSS Inc., Chicago, Ill. USA) was used. Continuous variables are expressed as mean ± SD. Group comparisons of the various parameters were performed by ANOVA with post-hoc comparisons (LSD) between specific groups where appropriate. Categorical variables are reported as percentages and were compared using a Chi square test. Correlations were tested by the Pearson 2-tailed correlation. Multiple regression analysis was used to test for independent correlations. All tests were performed at 95 % confidence intervals and a p-value of < 0.05 was considered statistically significant. Variability of measurements was assessed by the within-subject coefficient of variation (calculated as ratio of the SD of the measurement difference to the mean value of all measurements, expressed as a percentage).

## Results

### Study population

Demographic and 2D echocardiographic characteristics of the patient cohorts are summarized in Table 1. Barlow patients were slightly younger than subjects in the other two groups. Left ventricular systolic function was within the normal range in all three groups. The size of the left ventricle did not differ between patients and controls. The left atrium was significantly enlarged in MR patients, without any significant difference between the patient cohorts. The prevalence of flail leaflets was much higher in FED compared to Barlow patients. Mitral cleft was also more common in the FED group.

**Table 1:** Patient characteristics

	Barlow (*n* = 27)	FED (*n* = 32)	Control (*n* = 18)
Age (years)	51 ± 14^a^	62 ± 12	57 ± 16
Woman (n, %)	10 (37 %)	13 (41 %)	7 (39 %)
BSA (m^2^)	1.90 ± 0.19	1.95 ± 0.23	1.87 ± 0.24
Hypertension	13 (48 %)^a^	26 (82 %)^b^	10 (57 %)
Diabetes	3 (11 %)^b^	3 (9 %)^b^	0
EF (%)	66.2 ± 5.8	67.1 ± 6.1	65.4 ± 5.3
LV EDV (ml)	133.1 ± 49.9	128.2 ± 40.8	105.5 ± 29.4
LV ESV (ml)	44 ± 18.4	43.9 ± 12.7	37.5 ± 16.5
LAVi (ml/m^2^)	52.7 ± 13.7^b^	57.7 ± 29.5^b^	36.9 ± 8.6
PAPs (mm Hg)	38 ± 8^a,b^	47 ± 16^b^	29 ± 5
MR (n, %)			
None	0^b^	0^b^	6 (33 %)
Mild	0^b^	0^b^	11 (61 %)
Moderate	11 (41 %)^a,b^	2 (6 %)	1 (6 %)
Severe	16 (59 %)^a,b^	30 (94 %)^b^	0
Flail	10 (37)^a,b^	26 (81 %)^b^	0
Cleft	6 (22 %)^a,b^	12 (38 %)^b^	0

*BSA* indicates body surface area, *EF* ejection fraction, *LV EDV* left ventricular end diastolic volume, *LV ESV* left ventricular end systolic volume, *LAVi* left atrial volume indexed to BSA, *PAPs* systolic pulmonary artery pressure, *MR* mitral regurgitation

^a^indicates significant difference between the Barlow and FED groups

^b^indicates significant difference compared to control

### Geometry of the mitral apparatus

End-systolic static geometric parameters of the mitral annulus, including anteroposterior and transverse diameters as well as 2D and 3D annular areas were enlarged in both patient groups, with Barlow patients displaying larger values in all cases. Annular dilatation was proportionate in both patient groups, with similar ellipticity values in all three cohorts. Although no intergroup differences in the absolute values of annular height were observed, Barlow patients displayed relatively flatter annuli as evidenced by a significantly reduced AHCWR. This was concordant with decreased non-planarity in both patient cohorts, with Barlow patients displaying significantly higher non-planar leaflet angles.

Regarding valvular parameters, as expected, 3D leaflet area was increased in myxomatous patients with significantly higher values in the BD group. Patients with Barlow morphology also showed significantly larger prolapse height and volume compared to the FED group. Static geometric parameters of the three study groups are summarized in Table 2.

**Table 2:** Static annular and valvular geometric parameters

	Barlow (*n* = 27)	FED (*n* = 32)	Control (*n* = 18)
Annular parameters			
Antero-posterior diameter (mm)	37.3 ± 6^a,b^	34.4 ± 3.4^b^	30.9 ± 3.5
Transverse diameter (mm)	46.6 ± 5.0^a,b^	43 ± 4.1^b^	37.7 ± 4.0
3D mitral annular area (cm^2^)	15 ± 2.8^a,b^	13.3 ± 2.4^b^	10.6 ± 2.3
Ellipticity index (%)	126 ± 13	125 ± 10	117 ± 29
Non-planar angle of leaflets (°)	151.6 ± 16.4^a,b^	151.7 ± 12.7^b^	147.2 ± 12.4
Annular height (mm)	4.2 ± 1.5	3.7 ± 1.4	4.0 ± 1.0
AHCWR	13.1 ± 4.1^b^	13.9 ± 5.6	15.7 ± 3.7
Valvular parameters			
3D total leaflet area (cm^2^)	18.5 ± 4.9^a,b^	14.3 ± 2.9^b^	11.3 ± 2.7
Prolapse height (mm)	7.5 ± 2.8^a,b^	5.8 ± 2.6^b^	0.8 ± 0.6
Prolapse volume (ml)	3.8 ± 2.9^a,b^	1.2 ± 1.3^b^	0.0 ± 0.1

Data are based on measurements on an end-systolic frame. Values are presented as mean ± SD

^a^indicates significant difference between the Barlow and FED groups

^b^indicates significant difference compared to control

### Dynamic behaviour of the myxomatous valve in systole

**Fig. 2:**
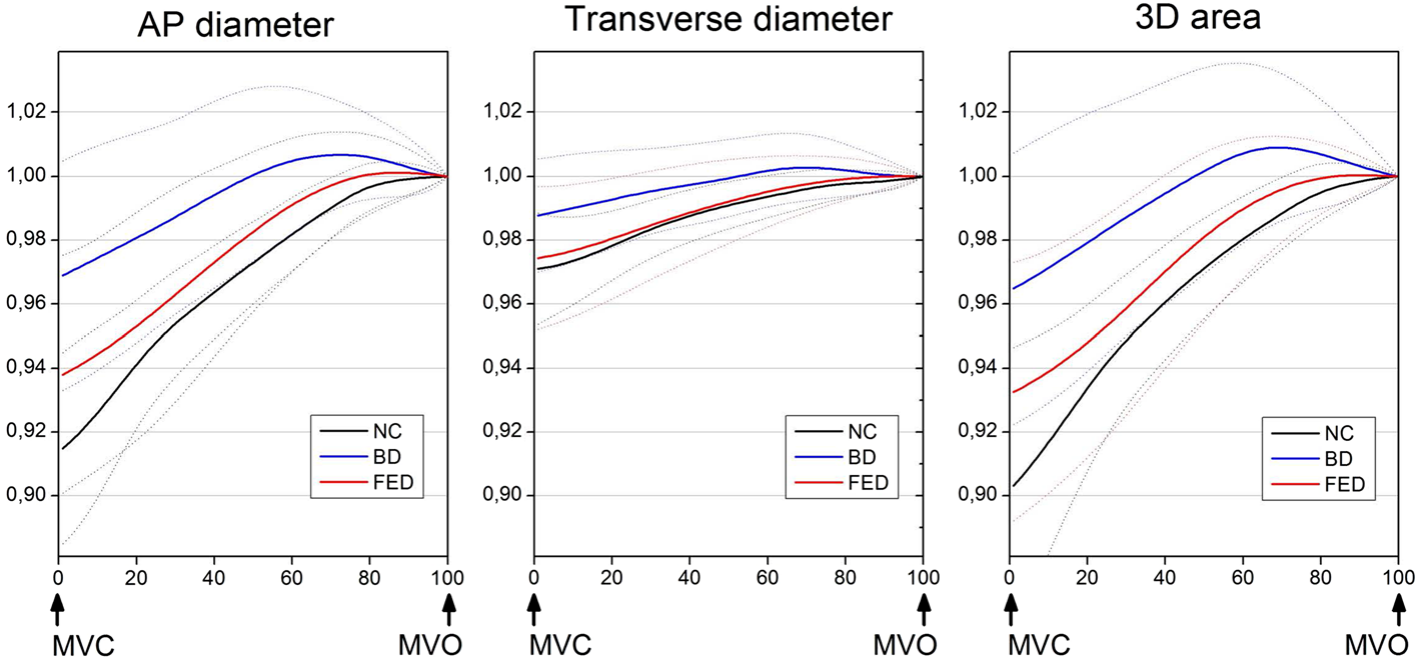
Dynamic behavior of the mitral annulus in systole. AP diameter, transverse diameter and 3D area changes of the mitral annulus are displayed over time. Continuous parameters are normalized for their end systolic value. Bold lines indicate mean values, dotted lines indicate SD

**Fig. 3:**
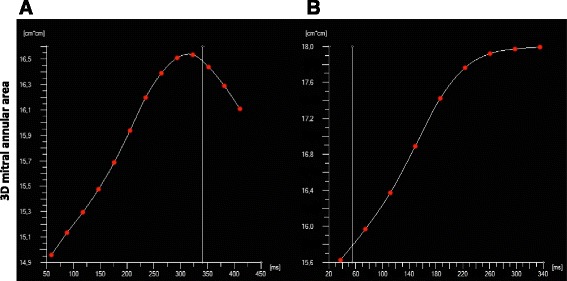
Dynamic model of the mitral valve in Barlow disease (**a**) and FED (**b**) produced by the TomTec software. The graph represents the 3D annular area over time

Three-dimensional dynamic models were created for all patients. Systolic changes of the annular geometric parameters over time are illustrated in Fig. 2. In all cases, a gradual increase of the 3D area and both the AP and transverse diameters were observed until end systole. Myxomatous annuli appeared less dynamic than normal, with decreased overall 3D area change, however only the BD group differed from NC significantly (BD vs. FED vs. NC relative 3D area change 4.40 vs. 6.81 vs. 9.69 %; BD vs. NC *p* = 0.001; FED vs. NC *p* = NS, BD vs. FED *p* = 0.025). The annuli of BD patients displayed significantly reduced shrinking along both axes, while in FED patients, dynamic changes along the transverse diameter were preserved with mildly restricted contraction along the AP diameter. Interestingly, despite the evident overall hypodynamicity of the diseased annuli, in Barlow patients, towards end-systole, a pathological overstretching of the MA beyond its end-systolic dimensions was also observed (Fig. 3a, b). The annular 2D area fraction change ((area_max_–area_min_)/area_max_), was significantly reduced in both patient cohorts. Although hypodynamicity in BD was more pronounced than in FED, the difference was not statistically significant (BD vs. NC 2D area fraction change 7.2 ± 4.5 vs. 12.2 ± 5.3 %, *p* < 0.01; FED vs. NC 8.9 ± 4.6 vs. 12.2 ± 5.3 %, *p* < 0.05; BD vs. FED *p* = NS). In order to investigate any structural determinants of the restricted motion of myxomatous annuli correlations between the static geometric parameters, including 3D annular area, AP and transverse annular diameters, AHCWR, 3D leaflet area, prolapse volume, prolapse height, and annular 2D area fraction change was tested. Multiple regression analysis failed to identify any geometric parameters as significant confounders (*p* = NS in all cases).

### Reproducibility

All measurements were performed by a single investigator. Intra-observer measurement variability for static annular geometric parameters was: 1.5 % for the antero-posterior diameter; 2.0 % for the transverse diameter; 1.1 % for the 3D mitral annular area and 3.1 % for the annular height.

## Discussion

In the present study we set out to selectively investigate distinctive morphological and dynamic characteristics of the mitral apparatus in the two major forms of MMVD, Barlow disease and FED. Our results are in agreement with data from previous studies, in finding that myxomatous annuli and leaflets are significantly enlarged, as demonstrated by various 3D geometric parameters (3D annular area, AP diameter, transverse diameter, 3D leaflet area) [5, 10, 11]. Significant differences within the myxomatous group are present regarding the degree of annular dilatation and prolapse, as described by leaflet area, prolapse height and volume, indicating more extensive lesion in BD. Ellipticity of the MA was preserved in our patients, in contrast to other studies that reported decreased annular ellipticity in MMVD, similarly to the circular annular remodeling observed in FMR. [10–12] The characteristic saddle shape of the MA, essential for optimal leaflet coaptation and minimization of leaflet stress [13], was significantly flattened in both forms of MMVD, with more pronounced deformation in the Barlow cohort. Both of these findings are confirmed in a recent study by Clavel and collegues, who found similar 3D annular morphology in myxomatous patients [14].

Regarding functional alterations of the MA in MMVD, the limited data available in literature are inconsistent. Some investigators report reduced [5, 15], others increased annular dynamics in MMVD patients [12, 16, 17]. Physiologically the 3D area of the mitral valve is maximally contracted in protosystole and continuously dilates during systole. We demonstrate significant difference in annular dynamics between the two forms of MMVD. According to our results the protosystolic contraction of the MA in FED patients resembles normal, however, it is significantly reduced in the BD group. In addition, Barlow patients display an abnormal contraction pattern with markedly reduced shortening along the transverse diameter by the end of diastole and pathological expansion in both directions during end-systole. Only one study has comparatively investigated the dynamic behaviour of the MA in the distinct forms of MMVD [14]. In their patient cohort, a slightly different annular motion patter was observed. The transverse diameter in FED patients, as opposed to the near-normal contraction in our study, remained stable throughout the cardiac cycle. In the Barlow group, over-expansion of the Barlow annuli was observed along the transverse diameter only, which displayed a continuous enlargement, without a protosystolic contraction. Notably, no data from healthy controls were analyzed in that report, therefore the extent of hypodynamicity as compared to normal could not be directly assessed [14].

Novel information available by the evolvement of 3D TEE technology [18] inspired the design of an increasingly wide range of annuloplasty rings and bands, developed with the intention to achieve more physiological postoperative conditions. Rigid and complete rings reliably prevent annular dilation, in addition, saddle-shaped rings restore physiological annular geometry; however, these have a negative impact on LV function and annular dynamics. Conversely, although partial and semi-rigid or flexible devices allow more normal annular motion, they run a higher risk of continued annular dilation, thus poor durability and recurrent MR.

A number of studies have investigated, the effect of various devices on annular shape and dynamics, with conflicting results [19–23]. More importantly, in terms of clinical outcome, no clear difference between various solutions have been shown [24, 25]. These results question the rationale behind the use of more physiologic ring implants. However, most of the aforementioned studies had several methodological shortcomings. Most importantly, the patient populations were inhomogeneous with respect of the etiology of mitral regurgitation, thereby ring selection has not been based on individual preoperative properties of the mitral apparatus in terms of 3D geometry and dynamics. Undoubtedly 3D analysis improves our understanding of the physiologic characteristics and pathological alterations of the mitral apparatus. However, this knowledge will not translate into improvement in terms of surgical results until the obtained information is actually used to govern therapy. Little of the data available by modern imaging technology is really used in clinical decision making. Possibly the failure to demonstrate a superiority of one type of ring over another does not reflect erroneous device design concepts, but rather inadequate device selection in individual cases. The solution might lie on optimally matching the individual properties of the diseased mitral apparatus in each patient with the best surgical solution [26].

Based on our results, we propose that due to their distinct geometric and functional characteristics, the different forms of myxomatous MVD might require different evaluation with respect to the choice of annuloplasty ring. In case of BD, where the contraction of the mitral annulus is reduced, the use of a costly flexible ring might not be beneficial, whereas in case of FED it might be important for preserving annular dynamics.

Certain limitations can be appreciated in this study. It was performed in a retrospective manner on a limited number of patients and control subjects – still, ours is the largest study investigating MV dynamics in myxomatous patients. The enrollment of patients into one or the other myxomatous group was based on established echocardiographic criteria^2^ and as only a proportion of patients underwent surgery, surgical inspection did not in all cases confirm the classification. The applied analysis software enabled investigation of the systolic phase of annular motion only. Most publications describe maximal annular contraction in protosystole, although some investigators report it in pre-systole. Regarding the limited temporal resolution of the applied RT3DTEE technique, it is possible that the time-point when the mitral annulus is at its minimum area was not included in the time interval marked as systole. However, as the same method was employed for all participants the results are not expected to be affected in terms of statistical differences between the groups. Patients with permanent atrial fibrillation were not analysed in this report. Although atrial fibrillation is a frequent finding in patients with severe MR, the reason for excluding these patients is that the presence of long standing atrial fibrillation itself results in significant chamber and annular remodeling as well as abnormal annular function [27, 28]. Therefore we considered that the analysis of the effect of myxomatous disease on annular geometry and dynamics is more appropriate in patients in sinus rhythm.

## Conclusions

We conclude that the two main forms of MMVD, BD and FED, differ significantly from each other with respect to the morphological and functional features of the mitral apparatus. Although both forms are characterized by annular dilatation, flattening of the characteristic saddle shape and hypodynamicity of the annulus is pronounced in BD only, whereas in FED the 3D non-planar shape and the contractile function is relatively preserved. These features may importantly influence the choice of repair technique and selection of annuloplasty ring, if surgery is necessary. Further studies are warranted to demonstrate whether a tailored ring selection governed by the above described considerations results in better surgical results and clinical outcome.

## Abbreviations

AHCWR, mitral annular height to commissural width ratio; AP, anteroposterior; BD, Barlow disease; BSA, body surface area; EF, ejection fraction; FED, fibroelastic deficiency; LAVi, left atrial volume indexed to BSA; MA, mitral annulus; MMVD, myxomatous mitral valve disease; MR, mitral regurgitation; NC, normal control; RT3DTEE, real-time 3-dimensional transesophageal echocardiography; TEE, transesophageal echocardiography
